# Misdiagnosis of Frontotemporal Dementia in a Patient With Progressive Supranuclear Palsy: A Case Report and Volumetric Neuroimaging Application

**DOI:** 10.7759/cureus.81421

**Published:** 2025-03-29

**Authors:** Camila B Eguiguren, Mateo D Fabara, Amelia Diaz

**Affiliations:** 1 Medical School, Universidad Internacional del Ecuador, Quito, ECU; 2 Medical School, Universidad de las Américas, Quito, ECU

**Keywords:** clinical case report, neurodegenerative disorders, neuroimaging findings, neuropsychiatric manifestations, progressive supranuclear palsy

## Abstract

Progressive supranuclear palsy (PSP) is a tauopathy, considered a movement disorder in which the clinical features can vary through the course of the disease. Some patients could manifest motor dysfunction since the onset of the disease, while others could present with psychiatric symptoms; therefore, PSP may be misdiagnosed as a different type of atypical parkinsonism, as a psychiatric disorder, or as frontotemporal dementia due to their similar clinical manifestations and basic neuroimaging characteristics. Here we present the case of a patient with PSP whose diagnosis was delayed due to an incorrect clinical assessment and a lack of proper neuroimaging analysis. This is the first reported Ecuadorian case of a 63-year-old male with PSP, that initially presented with psychiatric symptoms of impulsivity, depression and a suicide attempt before any motor symptoms were identified. The condition was initially misdiagnosed as frontotemporal dementia and treated accordingly, delaying the correct diagnosis and management. In this case, we review the pathophysiology of psychiatric symptoms and currently available neuroimaging techniques. To assure the accuracy of this report, CARE guidelines were followed and neuroimaging analysis was carried out using the ITK-SNAP software. This case aims to highlight the benefit of an early and accurate diagnosis with the assistance of volumetric MRI analysis and discuss the pathophysiology of PSP and its relationship with neuropsychiatric manifestations as an early sign of the disease.

## Introduction

Progressive supranuclear palsy (PSP) is a rare neurodegenerative disorder characterized by the abnormal accumulation of the tau protein leading to neuronal dysfunction and gliosis [1]. PSP has a prevalence of 6.2-7.4 per 100,000 people [2]. The classic phenotype known as Richardson syndrome (PSP-RS) includes motor symptoms such as bradykinesia, impaired gait, postural instability, early falls in the clinical course, and vertical supranuclear gaze palsy [3]. These last two symptoms are essential to diagnose a probable PSP, however, oculomotor findings take an average of three to four years to develop after the onset of the disease [4]. The clinical spectrum of PSP has extended beyond PSP-RS, leading to the identification of multiple phenotypes such as PSP-parkinsonism (PSP-P), PSP-frontal (PSP-F), PSP-corticobasal syndrome (PSP-CBS), PSP-speech/language variant (PSP-SL), and PSP-gait freezing (PSP-PGF), each defined by its predominant features [5]. This expanded classification showcases the myriad of presentations in a patient with PSP and highlights why misdiagnosis is common, as clinical characteristics of PSP overlap with other dementias and movement disorders, such as frontotemporal dementia (FTD). It remains a challenge to diagnose PSP, as both PSP and FTD share clinical symptoms including: apathy, impulsive behavior, cognitive decline, language disorders such as dysarthria and in severe cases, mutism, as well as motor symptoms like rigidity, bradykinesia, and postural instability [3]. To improve diagnostic accuracy, neuroimaging techniques play a crucial role, where midbrain volumetric measurements with magnetic resonance imaging (MRI) have demonstrated a high sensitivity and specificity to establish the diagnosis [6]. Furthermore, a proper clinical assessment is necessary, including a complete battery of neurocognitive and neuropsychiatric assessments.

In addition to motor dysfunction, neuropsychiatric manifestations are frequently observed in PSP affecting approximately 50 to 77% of patients throughout the course of the disease [7]. However, only 2.6% of patients present psychiatric symptoms at disease onset, as reported in a cohort of 187 patients [8]. To the best of our knowledge, we present the first Ecuadorian case of PSP, where mild depressive symptoms preceded a suicide attempt, initially leading to a misdiagnosis of major depressive disorder and frontotemporal dementia and, where advanced volumetric MRI analysis aided in the correct diagnosis of the disease.

## Case presentation

A 63-year-old married male orthopaedic surgeon, with a history of mild depressive symptoms but no prior suicidal ideation, presented to the emergency department in February 2023 following an intentional benzodiazepine overdose. After initial recovery, the neurological assessment revealed no motor deficits and clinical history showed no relevant personal or family antecedents. Upon further psychiatric evaluation, the patient did not exhibit suicidal ideation but displayed apathy, irritability, lack of empathy, and impulsive behaviour such as compulsive shopping and destroying personal belongings. At the time, the only neurological finding was a positive glabellar sign, while motor function, oculomotor movements, coordination, and gait were preserved.

Given these clinical findings, in March 2023 an MRI was performed to investigate potential underlying neurological conditions. The scan revealed frontotemporal and parietal atrophy, leading to a misdiagnosis of frontotemporal dementia. At that time the patient was treated with donepezil and mirtazapine for depressive symptoms, and neurocognitive tests were conducted, as shown in Table 1. By September 2023, the patient exhibited motor slowing, writing difficulties, and mild gross motor impairments in activities such as driving and dancing. Neurological examination revealed cogwheel rigidity, although no other neurological functions were impaired. A follow-up neurocognitive assessment was conducted in October 2023 (Table 1).

**Table 1:** Neurocognitive assessments MMSE: Folstein Mini-Mental State Examination, FAB: Frontal Assessment Battery, MIS: Memory Impairment Screening, WCST: Wisconsin Card Sorting Test, TMT: Trail making test, T@M: Memory Impairment Test. The general conclusions of the assessments were MMSE: improvement of global cognitive function. FAB: improvement in executive function and frontal control. MIS: no changes in short-term memory. WCST: improvement in cognitive flexibility. Stroop: no changes in inhibition capacity. TMT: improvement of speed processing. T@M: preserved episodic memory function.

	MARCH 2023		OCTOBER 2023	
Test	Score	Interpretation	Score	Interpretation
MMSE	26/30	Cognitive deterioration	30/30	Normal
Rey Complex Figure Copy	22/36	Preserved	20/36	Preserved
FAB	14/18	Altered	16/18	Preserved
MIS	6/8	Preserved	6/8	Preserved
WCST	4/6	Altered	6/6	Preserved
Stroop Test	–	Preserved	–	Preserved
TMT	–	Part A Preserved, Part B Altered	–	Part A Preserved, Part B Preserved
T@M	45/50	Preserved	45/50	Preserved

**Figure 1:**
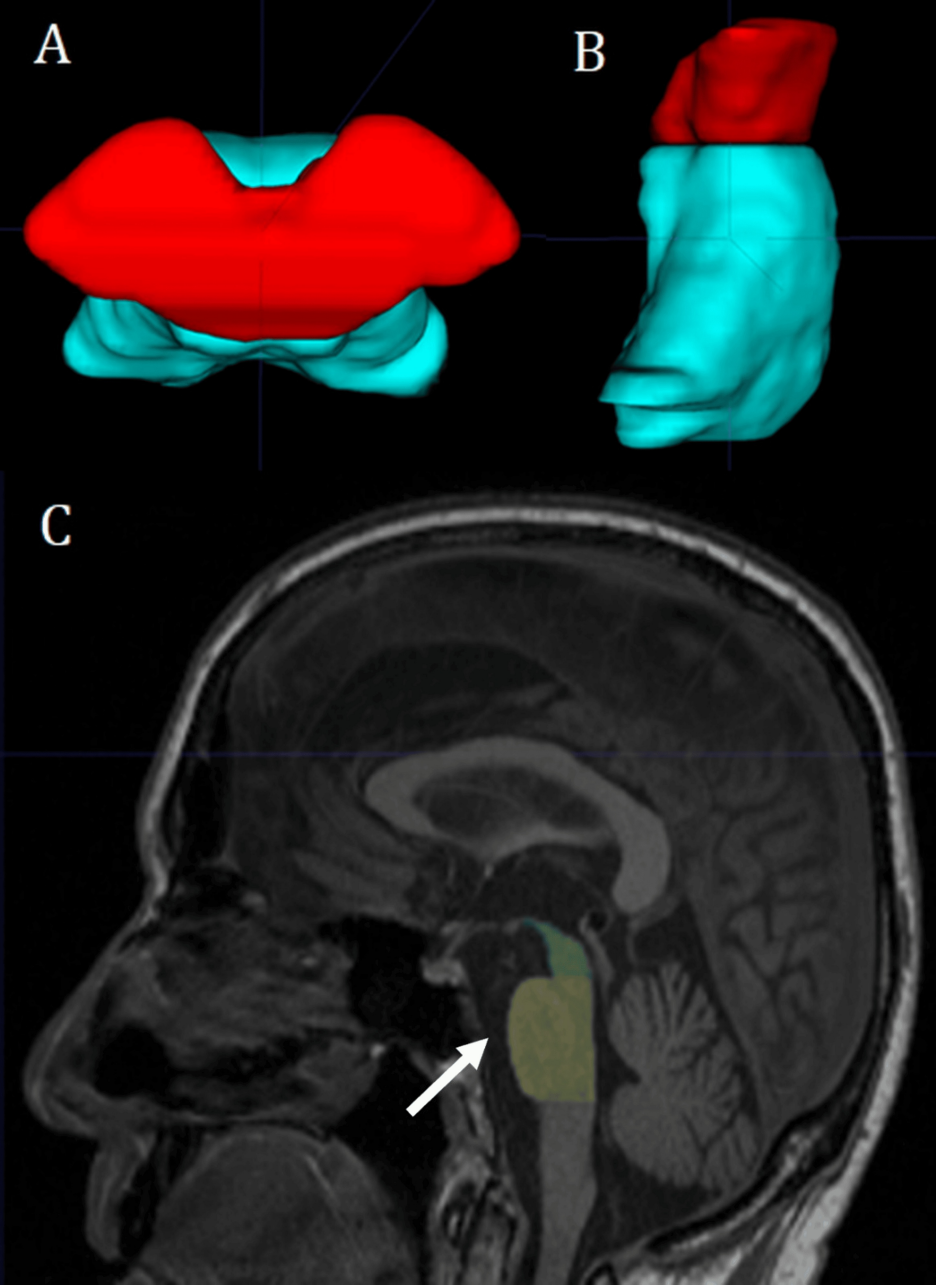
Volumetric and Morphometric MRI Analysis Panel A shows the volumetric reconstruction, the midbrain is seen in red and the pons in turquoise (superior view). Panel B presents the lateral view of the reconstruction. Panel C shows a mid-sagittal T1 MRI image, the pons is labelled as yellow and the midbrain as green; note the characteristic “hummingbird sign”, named due to the thinning of the anterior aspect of the midbrain resembling the beak of a hummingbird (white arrow).

Concerned about disease progression, the patient’s family sought a second opinion on the MRI findings. Therefore, manual volumetric analysis and segmentation was conducted using the software ITK-SNAP (Version 3.8.0-beta) and the volume, area, and lengths of the midbrain, pons, superior and inferior cerebellar peduncles were measured to calculate the magnetic resonance parkinsonism index (MRPI) following the protocol established by Quattrone et al. [6]. The midbrain had an antero-posterior diameter of 8.65 mm (normal >17 mm), with an area of 100 mm^2^, and a volume of 4.66 cm^3^. The pons had an area of 496.3 mm^2^ and a volume of 14.2 cm^3^. The average width of the middle and inferior cerebellar peduncles was 8.41 mm and 2.94 mm, respectively. In addition to these calculations, midbrain atrophy was evident in the visual examination as the “hummingbird sign” was clearly seen on the mid-sagittal projections (Figure 1). Finally, the MRPI was calculated as 14.19, considering that the cut-off point for differentiating PSP from healthy individuals is an MRPI ≥13.58 [6].

**Figure 2:**
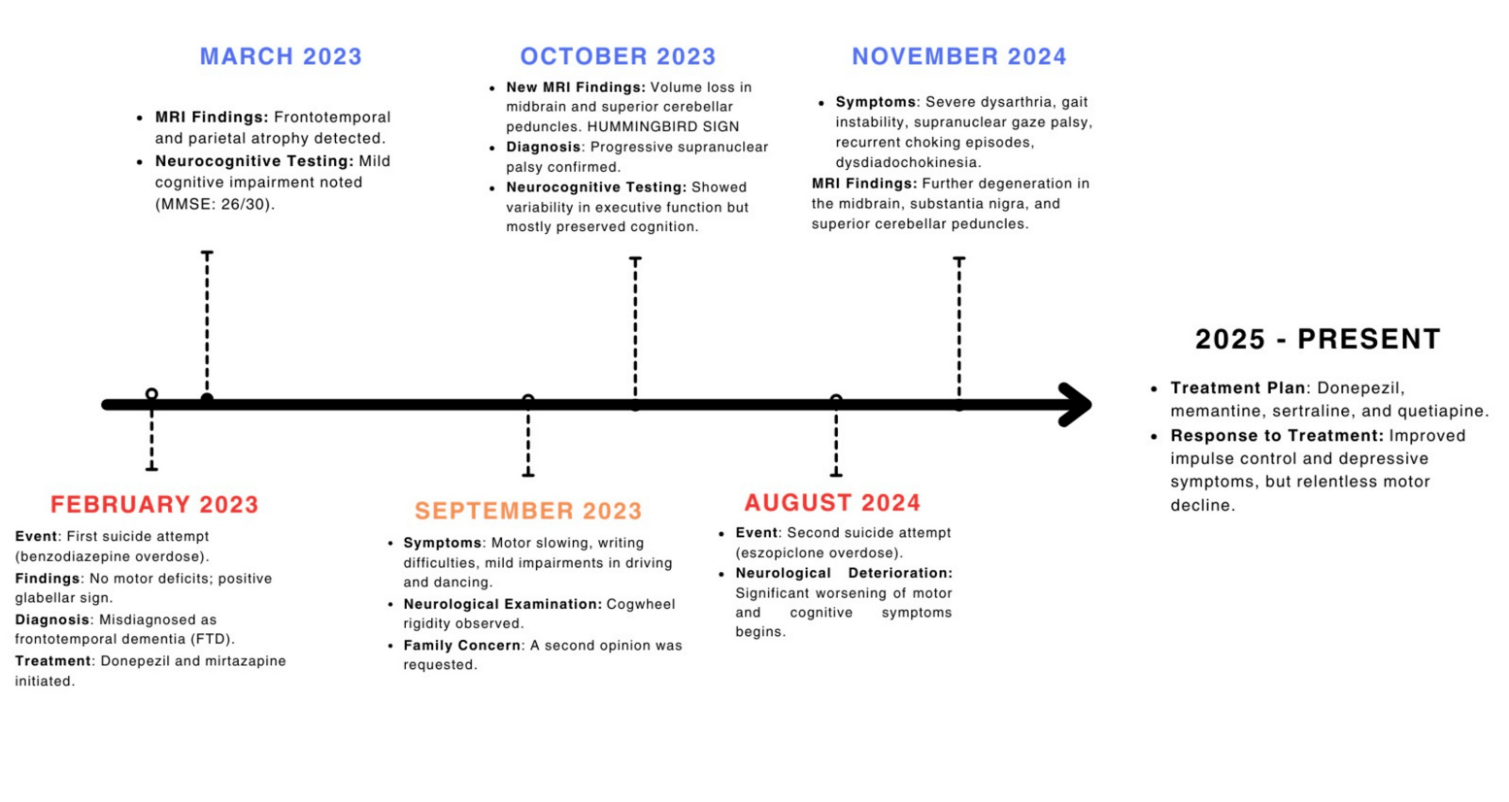
Clinical and Diagnostic Timeline Image Credits: Mateo D. Fabara

Approximately one year later, in August 2024, the patient attempted suicide for a second occasion during an impulsive episode, involving an eszopiclone overdose. This event marked the onset of significant neurological deterioration. A follow-up MRI revealed progressive degeneration in the midbrain and cerebellar peduncles. Between August and November 2024, the patient exhibited significant progression of both motor and cognitive symptoms, including severe dysarthria, recurrent episodes of dysphagia, gait instability, supranuclear gaze palsy and dysdiadochokinesia. A full timeline of events can be seen in Figure 2.

The patient is currently undergoing treatment with donepezil/memantine 10 mg/14 mg daily, sertraline 50 mg daily, and quetiapine (tapered from 100 mg to 25 mg daily) with appropriate adherence, tolerance, and no adverse reactions. While the impulse control and depressive symptoms have improved with treatment, motor decline remains relentless.

## Discussion

To our knowledge, this is the first reported case of progressive supranuclear palsy in Ecuador presenting psychiatric symptoms as the initial manifestation. Given the phenotypic variability of PSP, early recognition of neuropsychiatric symptoms is crucial for a timely diagnosis and intervention. However, the literature remains inconsistent regarding the percentage of patients presenting with psychiatric manifestations. On one hand, Nath et al. reported that 2.67% of 187 PSP patients presented with depression or apathy at disease onset [7], while other authors have found neuropsychiatric symptoms in 6.7% and 9% of their respective cohorts [4,9]. On the other hand, other authors have reported higher rates with a 42% prevalence of depression, as a pre-diagnostic feature [10]. This discrepancy highlights the need for further research to better understand the role of early psychiatric symptoms in PSP and their underlying pathophysiological mechanisms.

Current research suggests that an early dysfunction of subcortical circuits such as the orbitofrontal and medial frontal circuits could explain the disinhibition and apathy presented, respectively [11]. Furthermore, apathy has been linked to reduced grey matter volume in the right lateral ventral prefrontal cortex [11]. Furthermore, some inflammatory processes and oxidative stress may contribute to early psychiatric symptoms. Experimental studies in animal models have shown that infection-induced oxidative stress and NFκB pathway can trigger depressive-like behaviour [2]. Evidence suggests that mitochondrial dysfunction plays a role in PSP pathology, as alterations in complex I activity lead to increased oxidative stress, which in turn activates tau kinases, promoting tau hyperphosphorylation and aggregation [2]. This pathological process appears to particularly affect monoaminergic brainstem nuclei, potentially contributing to depressive symptoms through brainstem dysfunction [2]. These changes may be related to disruptions in neurotransmitter systems particularly dopaminergic and serotoninergic pathways [2]. It has been shown that striatal dopamine loss is severe in PSP, whereas serotonin levels in basal ganglia remain relatively preserved. However, the precise mechanisms by which these alterations contribute to the early psychiatric symptoms in PSP remain unclear [2].

FTD (frontotemporal dementia) and PSP could share both clinical and imaging features leading to an inaccurate diagnosis. Clinically both are characterized by behavioural symptoms like apathy, loss of empathy and social disinhibition, features that our patient initially exhibited [12,13]. Moreover, from a neuroimaging perspective, both may show cortical atrophy in the motor cortex, frontal, and temporal lobes. However, midbrain atrophy is a crucial characteristic in PSP, though in certain cases FTD may also exhibit midbrain involvement, particularly affecting the fronto-pontine and temporo-pontine pathways [14,15]. In this sense, neuroimaging is a key component in differentiating PSP from other neurodegenerative disorders. In our patient, midbrain measurements were obtained using manual morphometric volumetry, following the MRI parkinsonism index, designed by Quattrone et al. [6].

To calculate this index, Quattrone et al. [6] used T1-weighted volumetric MRI sequences. Measurements were taken from the midbrain (M) area and pons (P) in a midsagittal image, the MCPs (middle cerebellar peduncle) in a parasagittal image, and the SCP (Superior cerebellar peduncle) in an oblique coronal view. Each measurement was obtained in consecutive sections and later averaged to better reliability. The MRPI was calculated using the formula [(P/M) x (MCP/SCP)], where P/M represents the pons-to-midbrain area ratio and MCP/SCP corresponds to the ratio of MCP to SCP width. The sensitivity, specificity and positive predictive value (PPV) were determined for this index with the significance level set at P < 0.05 for differentiating PSP from other Parkinsonian diseases.

Other authors have proposed that an accurate diagnosis through the use of a support vector machine could be achieved with a sensitivity of 85% and specificity of 95% [16]. Wattjes et al. [16] recommend a combination of support vector machines with visual analyses by using the classical Hummingbird or Mickey Mouse signs. Although other imaging techniques such as positron-emission tomography (PET) with (18F)AV-1451 tracer or DATSCAN have been explored, differentiating PSP from other disorders like Parkinson's disease remains challenging [14,17]. Considering that PSP has a mean survival of approximately six to seven years, an early and accurate diagnosis is essential [18]. In this context, we suggest that in developing countries where PET is not widely available or affordable, volumetric measurements represent a valuable alternative to an early and correct diagnosis and therefore a better-orientated and prompt treatment.

## Conclusions

Progressive supranuclear palsy (PSP) is a tauopathy that affects the midbrain and is often misdiagnosed due to its variable clinical presentation and progression. Differentiating PSP from other atypical parkinsonism disorders, such as frontotemporal dementia, remains a challenge, particularly in settings with limited access to advanced imaging. Here, we present the case of an Ecuadorian patient incorrectly diagnosed with frontotemporal dementia due to a psychiatric onset, later correctly identified as PSP through volumetric and morphometric MRI analyses. This report highlights the importance of considering neurodegenerative disorders as a potential diagnosis in elderly patients with early psychiatric symptoms and reinforces the use of volumetric measurements as a valuable tool for early diagnosis, especially in developing countries. Despite these insights, we consider that there is still a lack of specialized training among healthcare professionals in volumetric analysis. Therefore, this highlights the importance of having qualified professionals to standardize this diagnostic tool. Further research should explore the expanding role of artificial intelligence in the volumetric assessment of PSP, offering prompt and accurate management.
